# Modelling Fire Frequency in a Cerrado Savanna Protected Area

**DOI:** 10.1371/journal.pone.0102380

**Published:** 2014-07-23

**Authors:** Alfredo C. Pereira Júnior, Sofia L. J. Oliveira, José M. C. Pereira, Maria Antónia Amaral Turkman

**Affiliations:** 1 Divisão de Sensoriamento Remoto, Instituto Nacional de Pesquisas Espaciais, São José dos Campos, Brazil; 2 Centro de Estudos Florestais, Instituto Superior de Agronomia, Universidade de Lisboa, Lisboa, Portugal; 3 Centro de Estatística e Aplicações, Faculdade de Ciências, Universidade de Lisboa, Lisboa, Portugal; DOE Pacific Northwest National Laboratory, United States of America

## Abstract

Covering almost a quarter of Brazil, the Cerrado is the world’s most biologically rich tropical savanna. Fire is an integral part of the Cerrado but current land use and agricultural practices have been changing fire regimes, with undesirable consequences for the preservation of biodiversity. In this study, fire frequency and fire return intervals were modelled over a 12-year time series (1997–2008) for the Jalapão State Park, a protected area in the north of the Cerrado, based on burned area maps derived from Landsat imagery. Burned areas were classified using object based image analysis. Fire data were modelled with the discrete lognormal model and the estimated parameters were used to calculate fire interval, fire survival and hazard of burning distributions, for seven major land cover types. Over the study period, an area equivalent to four times the size of Jalapão State Park burned and the mean annual area burned was 34%. Median fire intervals were generally short, ranging from three to six years. Shrub savannas had the shortest fire intervals, and dense woodlands the longest. Because fires in the Cerrado are strongly responsive to fuel age in the first three to four years following a fire, early dry season patch mosaic burning may be used to reduce the extent of area burned and the severity of fire effects.

## Introduction

**Figure 1:**
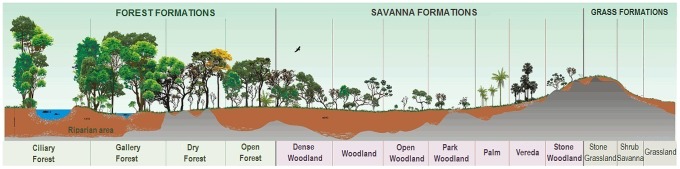
Physiognomies of the Cerrado. (adapted from [3]).

The Cerrado, the Brazilian tropical savanna, is a complex vegetation formation characterized by a mosaic of physiognomies ranging from pure grasslands through woodland to closed forest [1]–[3] (Figure 1). It is a biodiversity hotspot that covers ≈ two million km^2^ of central Brazil (about a quarter of the country area) and the second largest biome in South America, after the Amazon [4], [5]. The Cerrado is a fire-prone biome [1], [2], [6], [7] and anthropogenic wildfires have been playing an important role since at least the mid Holocene, from 6000 BP [8], [9]. Indigenous people used fire to manage their resources and the decision to burn was based on several ecological indicators, denoting a clear social control [10]. Yet, as at many other places in the world, current land use and agricultural practices have considerably changed fire regimes in the Cerrado, and at least 49% of the vegetation has been converted to extensive monocultures and pastures [11]. Furthermore, 80% of the biome has been disturbed and most of the natural remnants are now located in the northern limit of its distribution [4], [5]. This loss is twice the area lost in the Amazon forest and only 7.4% of the biome is inside protected areas [11].

Farmers use areas of the Cerrado for intensive farming, mostly soybean, but also corn, rice, cotton and sugarcane, and grass formations as natural pasture [2], [5]. In the dry season, this grassy herbaceous biomass becomes especially dry and very flammable and it is burned to promote fresh growth in natural pastures. Crop residues are also burned to clear harvest in preparation for planting at the beginning of the wet season [1], [2], [7], [12], [13]. The current fire regime is characterised by extensive and frequent anthropogenic fires, that occur most often during the middle and latter months of the 6-month dry season (April to September), under severe fire weather conditions [1], [2], [6], [7], [12]–[14]. Fire return intervals are usually very short, from one to four years [1], [2], [7], [12], [13]. Such a fire regime favours herbaceous species and encourages the maintenance of the open Cerrado physiognomies, leading to soil degradation, overall biodiversity loss and an increased greenhouse gas emissions [1], [2], [7], [12], [13].

However, because the Cerrado is a fire-prone biome, periodical fires are important for the creation and maintenance of landscape structure, composition, function and ecological integrity [15]–[17]. Fire is an important land management tool in tropical savannas of Australia [18], [19] and Africa [20], [21], but its use to protect and promote biodiversity is very limited in the Cerrado, with the exception of Emas National Park [14]. Analysis of tropical savanna fire regimes in the presence of active prescribed burning programs [22], [23] indicates a reduction in intensity, size, and wildfire hazard with favorable ecological and greenhouse gas emission implications. Progress has been made in recent years to understand fire regimes and their effects in the Cerrado [1], [12]–[14], [24]–[25]. Satellite remote sensing makes it possible to survey recent burned area history over large ecosystems, providing useful data to analyse features of the savanna fire regime, such as fire frequency. Mapping burned area from satellite imagery has been undertaken since the 1980s in the Brazilian Amazon [26]–[30] and in the Cerrado [31]–[34].

Remote sensing combined with statistical modelling is a powerful tool to quantify the uncertainty associated with fire regime descriptors, as shown in northern Australia [35], [36] and South Africa [37]. According to [38], a statistical model is “a probability distribution constructed to enable inferences to be drawn or decisions made from data”. As such, a statistical model here is a probability distribution assumed for the random variable of interest, namely the fire intervals over a 12-year time series. In the Cerrado there are few studies of fire frequency [14], [39], and none addressing statistical modelling of fire return intervals. The choice of a particular model based on fitting statistical distributions is of great utility when working with fire interval data, because it provides rigorous quantitative understanding of the properties of fire regimes.

In this study, we analysed fire frequency over a 12-year time series (1997–2008) for Jalapão State Park, a protected area in the north of the Cerrado, based on burned area maps derived from Landsat imagery. Using a discrete lognormal model, we estimated fire interval, survival and hazard of burning distributions for different physiognomies of the Cerrado. This analysis is very useful for fire management and biodiversity conservation, and for estimating greenhouse gas emissions in protected areas.

## Methods

### Ethics Statement

Jalapão State Park is a protected area managed by the Instituto Natureza do Tocantins (NATURATINS). Fieldwork to develop the land cover assessment was undertaken in collaboration with NATURATINS, under the scope of a scientific-technical cooperation convention (registration no. 01.01.008.0/2006), and therefore no additional approval or permit was required. No protected species were sampled.

### Study area

**Figure 2:**
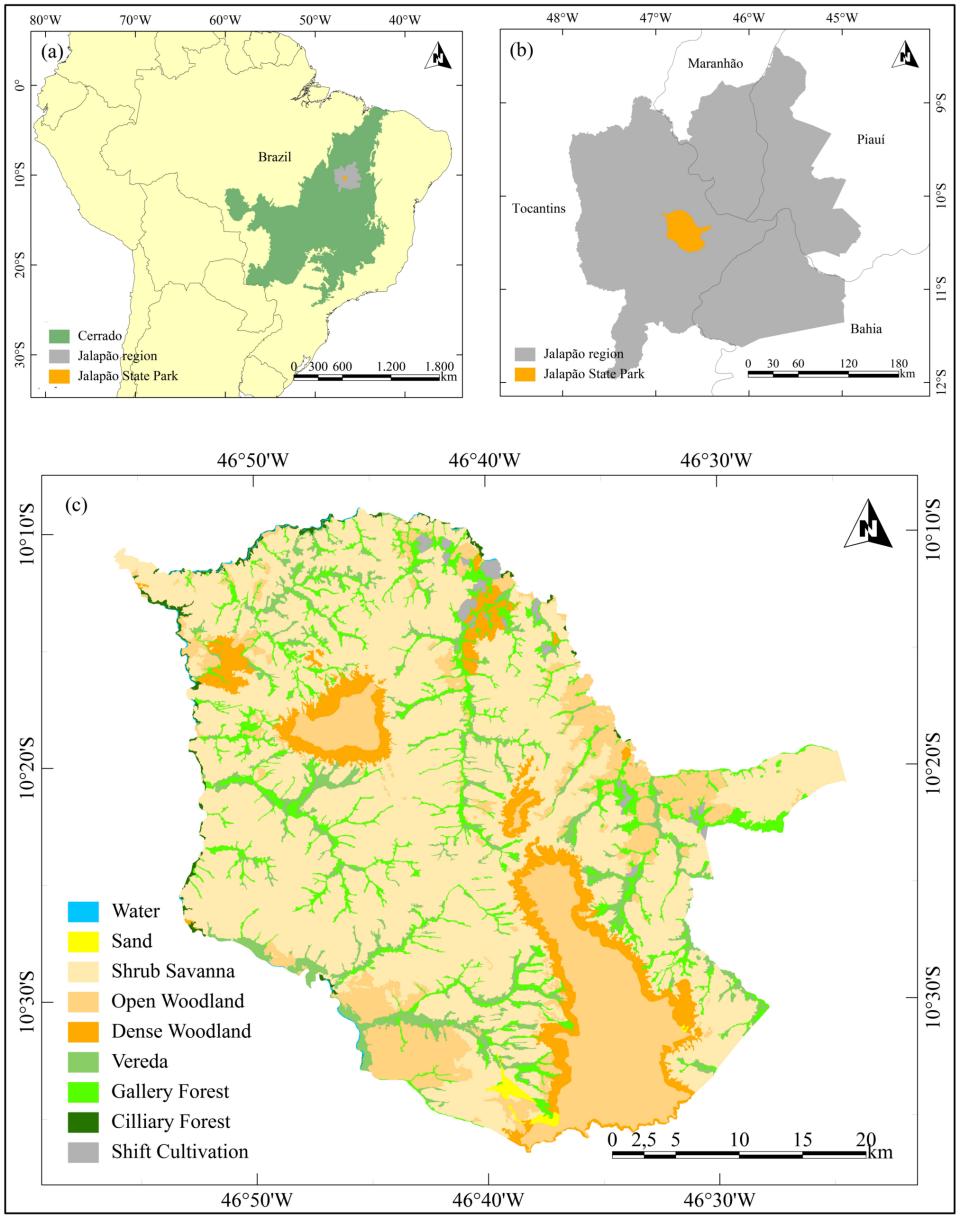
Location and land cover map of Jalapão State Park. (a) Cerrado biome (green) and Jalapão region (grey) in Brazil, (b) location of Jalapão State Park (orange) within Jalapão region (green), (c) land cover map adapted from the source map of [44] and modified based on fieldwork.

Jalapão State Park (JSP) was created in January 2001, with an area of 159,225 ha. It is located in Mateiros municipality, in the eastern part of Tocantins state, Brazil, between latitudes 10°08′53″S and 10°36′32″S and longitudes 46°24′24″S and 46°56′06″W (Figure 2). The park is located in the Jalapão region, which contains the largest officially recognized continuous preserved area of Cerrado in Brazil [40].

The climate at JSP is markedly seasonal (Aw in Köppen’s classification: Group A - tropical/megathermal climates, w - tropical wet and dry, or savanna climate) with high rainfall in the wet season (around 1500 mm), from October to March, and an extended dry season from April through September/October (only 10% of the annual precipitation). Year round temperatures are mild, ranging from 22° to 27°C [5]. Population density is approximately 0.6 inhabitants/km^2^, and the local economy is based on shifting cultivation, extensive cattle raising and, more recently, tourism and golden-grass (*Syngonanthus nitens, Eriocaulaceae)*) handcrafting [41], [42]. Fire is used in cattle ranching as a management tool to stimulate grass regrowth in the dry season, when forage is in short supply and to stimulate golden-grass flowering, for use in local handicrafts. Ranchers and handcrafters use biennial fires, but the absence of firebreaks allows fires to spread over larger areas than required by those land use practices [42].

Jalapão State Park is an agricultural frontier and an area attractive for tourism, two economic activities that generate pressures for land use change. The legal status of JSP as a protected area severely restricts land use, only allowing activities related to conservation, and forbids the use of fire. However, over 150 farmer and extractivist families inhabited the area prior to the creation of JSP, own their lands, and remain in situ. They engage in land use and resource management practices, namely vegetation burning, which often conflict with the nominal, feebly enforced JSP nature protection status. The conflict is deemed irreconcilable, unless the state purchases all private land within the park boundaries, relocates the resident populations, and protects the area from inappropriate use by non-residents [41], [42]. Only then, would it be possible to properly acknowledge the role of fire in Cerrado ecosystems and, rather than attempting full exclusion [15]–[17], implement fire management programs aimed at conserving and promoting biodiversity. The legal obligation to attempt fire exclusion has brought many problems to protected areas, such as disturbance of ecological processes, changes in species abundances, and biological invasions, which place the preservation of the biodiversity at risk, in the medium and long-term [15]–[19].

There are three main landscapes in JSP. First, the dryland on arenosol, where dominant physiognomies are Campo Sujo (Shrub Savanna - SS) and Cerrado Sensu Stricto (Woodland). The latter is divided into Cerrado Ralo (Open Woodland - OW), Cerrado Típico (Woodland - WD) and Cerrado Denso (Dense Woodland - DW) [3], [41]. Second, the wetland on arenosol along the watercourses, where physiognomies are Vereda (VR), Gallery forest (GF) and Cilliary Forest (CF). Third, the plateau, where Open Woodland (OW) occurs on flat surfaces of ferralsol, and Dense Woodland (DW) occurs on hills of leptosols [3], [41].

The Shrub Savanna (SS) is an herbaceous-shrubbery physiognomy with sparse shrubs over grassland. Woodland, divided into Open Woodland (OW), Woodland (WD) and Dense Woodland (DW), is a woody physiognomy with sparse trees and shrubs over grassland, where the woody layer is denser than in SS. Tree canopy cover in OW ranges from 5% to 20% and has an herbaceous-shrubbery layer denser than in WD and DW. Canopy cover in WD ranges from 20% to 50% and in DW ranges from 50% to 70% and both have a higher and more compact woody layer than OW [3]. Gallery Forest (GF) is a riparian physiognomy along narrow rivers, where the trees form a gallery over the watercourse with crowns overlapping and canopy cover from 70 to 95%. Belts of non-forest physiognomies surround both borders in an abrupt transition. Cilliary Forest (CF) is also a riparian physiognomy, present along medium and large rivers, where tree crowns do not form a gallery over the watercourse. Trees are predominantly deciduous and canopy cover ranges from 50 to 90% throughout the year. *Vereda* (VR) is a swampy area dominated by *buriti* palm (*Mauritia flexuosa,* Arecaceae) along narrow rivers (it can be associated with GF) and surrounded by belts of humid grassland [3], featuring golden-grass. Shifting cultivation (SC) is the agricultural system that occurs in JSP. It is defined as an “agricultural system that involves an alternation between cropping for a few years on selected and cleared plots and a lengthy period when the soil is rested” [43].

A land cover map of JSP was created by [44] using TM/Landsat-5 data acquired in 2007. The physiognomies WD and DW were not separable in this data and they were joined in one only class. This map was modified using field data collected in December 2005 and May 2008, that corrected errors existing in the source map, including land cover changes caused by fire (Figure 2c).

### Burned area mapping

Burned area maps for JSP were produced with data from the Thematic Mapper (TM) sensor on board the Landsat-5 satellite and the Enhanced Thematic Mapper plus (ETM+) on board Landsat-7, for the period 1997 to 2008. We used 47 Landsat images (path/row 221/67) that were downloaded from the U.S. Geographical Survey Global Visualization Viewer (glovis.usgs.gov) and from National Institute for Space Research (INPE) Remote Sensing Datacenter (dgi.inpe.br/CDSR). At least three images were acquired for each dry season: at the end of the early dry season (April to May); at end of the middle dry season (June to July); and at the end of the late dry season (August to September). This bi-monthly sampling regime reduced omission errors in the classification, because burn scars in tropical savannas ecosystems can disappear within a few weeks [14].

The Landsat based burned area classification was performed using Object Based Image Analysis (OBIA) with the software eCognition Developer 8.0 [45]. OBIA has previously been used to map burned areas using Landsat images with promising results [46], [47]. OBIA works in two steps: segmentation and classification. The segmentation splits an image into unclassified objects that are groups of pixels similar to one another based on a measure of spectral properties (i.e., color), size, shape, and texture, and pixel topology, controlled through parameters set by the user [48]. After segmentation, the image was classified by assigning each object to a class based on features and criteria set by the user. Segmentation and classification burned area outputs were considered final when they visually matched the original burned areas from the Landsat image. A minor amount of manual on-screen editing complemented the classification.

### Fire rotation period

The annual burned area maps from 1997 to 2008, obtained from OBIA classification, were overlain with the land cover map for JSP, using the software TerraView 3.3.1 [49]. Overall fire frequency at JSP can be quantified by the fire rotation period (FRP), which is the time needed to burn an area of the same extent as the study area [50]:
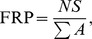
where *N* is the number of years in the study period; *S* is the area susceptible to burn in the study area; *A* is the annual area burned; and 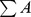 is the sum of *A* within the period *N*. The inverse of the FRP, the annual percentage of area burned (APAB), is the mean percentage of the study area burned each year. Fire frequency was calculated for all of JSP and by land cover type.

### Fire frequency analysis

Fire frequency analyses usually rely on the estimation of a time since fire (‘survival’) distribution and a fire interval (‘mortality’) distribution. These two distributions are linked by the hazard of burning function (force of mortality), which is the ratio of the mortality and survival distributions [51]. In tropical savanna ecosystems, the terms ‘survival’ and ‘mortality’ refer to a point or area having been unaffected or affected by fire. The most widely used statistical model in fire interval studies is the Weibull model [52], [53]. However, [36] recently showed that the discrete lognormal model was more appropriate for fire interval analysis in Australian tropical savannas. The discrete lognormal was chosen for this study to model fire intervals in the Brazilian Cerrado because it has fire frequencies and vegetation types similar to those of the Australian savannas.

A 500 m regular point grid was used to sample intervals between successive fires, by land cover class, using the time-series of burned area maps (1997–2008). Fire survival analysis deals with analysis of time duration to until one or more fires happen. We sampled complete fire intervals, when both the starting and ending date of the fire-free period were known, and single-censored intervals. Single-censoring is a form of missing data that occurs often in survival analysis when either the starting or the ending date of the fire-free period is unknown. Right censoring occurs for all vegetation patches whose date of origin is known, but that remain undisturbed when the study ends. If the vegetation patch age at the start of the study is unknown, its lifetime is said to be left-censored. Ignoring single-censored data tends to lead to incorrect fire frequency estimates [36], [53]–[55].

Fire interval data extracted with the point grid were used as input for the discrete lognormal model, to estimate fire interval, survival and hazard of burning distributions (e.g., [28], [52], [56]) using the survival package from software R [57]. The discrete lognormal model is a discrete version of a lognormal distribution, which is the distribution of a continuous random variable whose logarithm is normally distributed [58]. The fire interval distribution corresponding to the lognormal model, that is the probability that the time between consecutive fires is equal to *t*, is given by:
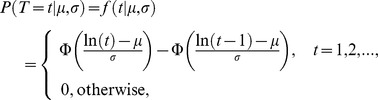
where σ and µ are parameters of the model, and Φ(*t*) is the distribution function of the standard normal distribution. The survival distribution is the probability of any given vegetation patch having survived fire longer than *t*;



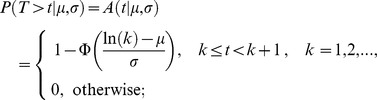



The hazard of burning distribution is a conditional probability of burning during time interval *t*, given survival up to that point:
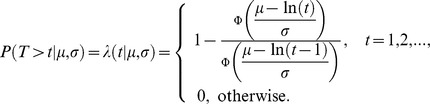



In general, this hazard distribution initially increases, then decreases eventually approaching zero. Hence, vegetation patches following a discrete lognormal model have higher chance of burning as they age for some period of time, but after survival to a specific age (turning point), the probability of burning decreases as time increases. This turning point depends simultaneously on the ratio *µ*/*σ* and on the value of *σ*, in a nonlinear fashion [36]. If the turning point is one, the hazard function is always decreasing. The discrete lognormal parameters (*σ* and *µ*) were estimated with a maximum likelihood approach. The median fire interval is the smallest integer that is bigger than or equal to exp (*µ*) and the mode of the distribution is the smallest integer that is bigger than exp (*µ* − *σ* ^2^).

A goodness-of-fit test for censored data developed by [59] was used to test the fit of the discrete lognormal distribution to the data. The test statistic, conveniently standardized, follows under the null hypothesis a standard normal distribution. Hence rejection of the proposed model is for values of the standardized test statistic above or below the adequate quantile of the standard normal distribution.

## Results

### Burned area mapping

**Figure 3:**
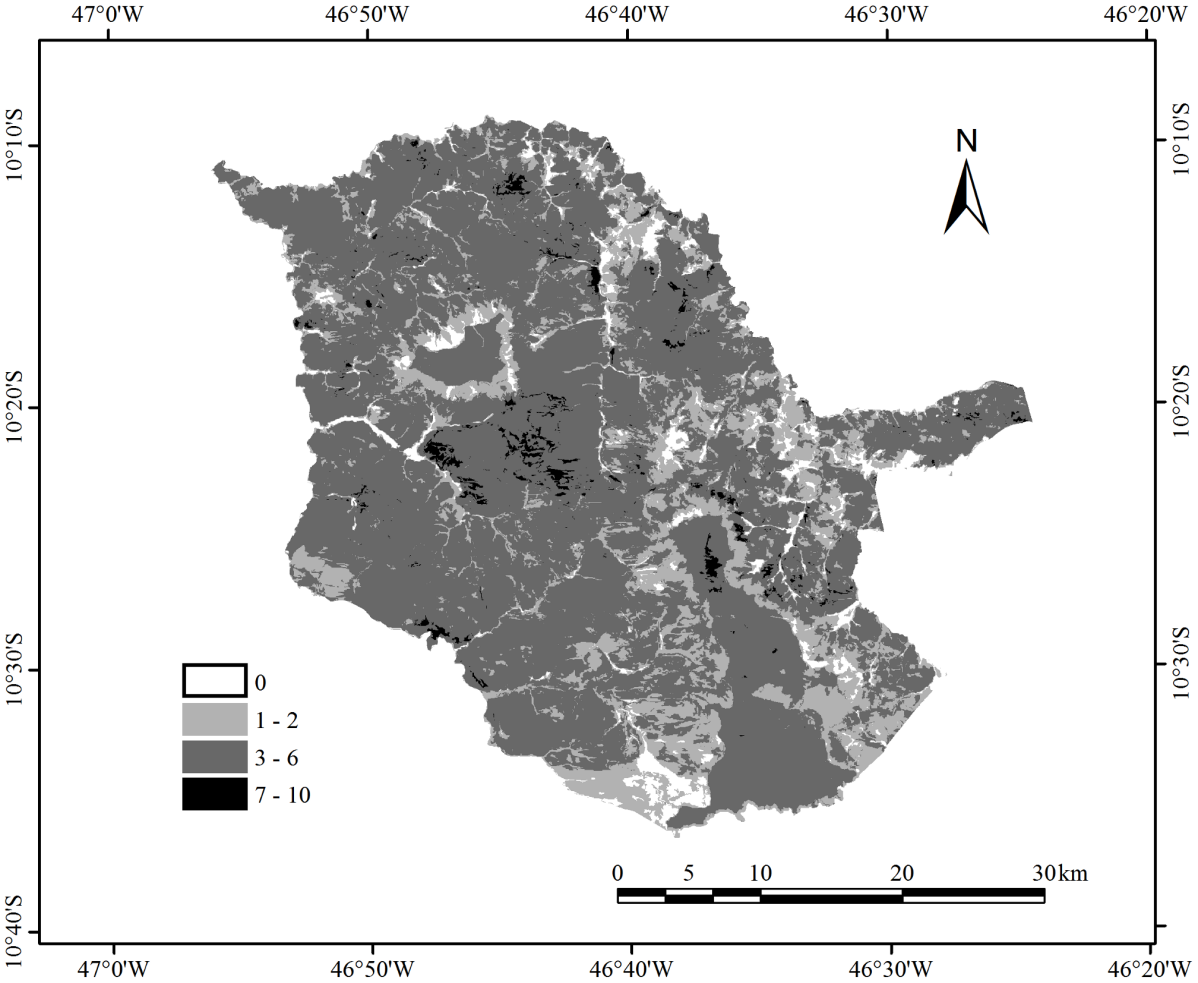
Number of times burned in Jalapão State Park, between 1997 and 2008.

The total area burned in JSP over the period 1997–2008 was 585,122 ha, which is equivalent to almost four times the size of the study area. The vast majority of the area affected by fire (77.6%) burned between three and six times (Figure 3). Only 6.4% of the area burned more than seven times and 16.0% burned once or twice. Unburned areas correspond to sand dunes and water bodies, and some areas of GF, CF and DW.

### Fire rotation period

The overall estimated FRP for JSP was 3.0 years and the APAB was 34%. However, values varied with land cover type (Table 1). The lowest FRP occurred in SS and OW (2.72 years and 2.75 years, respectively). SS and OW occupy 77.6% of the study area (59.1% and 18.5%, respectively) and accounted for 84.2% of the total area burned during the study period (64.3% and 19.9% respectively). The highest FRP, 5.49 years, was observed in DW, that occupies 6.9% of the study area and accounted for 3.7% of the total area burned). Intermediate values were found in swampy (VR) and riparian physiognomies (GF and CF) and in SC (Table 1).

**Table 1:** Fire frequency, fire interval descriptors, and goodness-of-fit tests, by land cover type for Jalapão State Park.

Land cover class	Area		FRP	APAB	*µ*	*σ*	Median	Mode	turning point	HP	p-values
	ha	%									
Shrub Savanna (SS)	93,680	58.9	2.72	37	0.82	0.66	3	2	4	−1.24	0.21
Open Woodland (OW)	29,310	18,4	2.75	36	0.93	0.69	3	2	4	−1.07	0.29
Dense Woodland (DW)	11,008	6.9	5.49	18	1.78	0.99	6	3	4	−0.90	0.37
Vereda (VR)	8,794	5.5	3.02	33	0.90	0.80	3	2	3	−1.03	0.31
Gallery Forest (GF)	13,459	8.5	4.44	23	1.19	0.91	4	2	3	−0.85	0.40
Cilliary Forest (CF)	908	0.6	4.57	22	1.39	0.92	5	2	4	−0.95	0.35
Shift Cultivation (SC)	1,233	0.8	3.71	27	0.86	0.89	3	2	3	−0.92	0.36

Other land cover classes not listed are sand (511 ha –0.3%) and water (250 ha –0.2%). Fire rotation period (FRP), annual percentage of area burned (APAB), estimated parameters µ and σ for the discrete lognormal model, median, mode and turning point, that is, the value where the hazard function attains its maximum. Hollander and Prochan (HP) test statistics provides a measure of the goodness of fit of the statistical model. P-values for the HP goodness of fit test are also shown. Since the p-values are above 0.05, there is statistical evidence to support the lognormal model.

### Fire frequency analysis

Estimated parameters for the lognormal model are shown in Table 1. The goodness-of-fit test never rejects the lognormal model, as shown by the test statistic and the corresponding p-values in Table 1. The turning point is larger than one for all land cover types, meaning that the hazard function follows the general pattern, increasing initially and then decreasing. Median fire intervals, can be aggregated in three groups of land cover: SS, OW, VR and SC with the lowest values (three years); GF and CF with intermediate values (four and five years); and WD with the highest median fire interval (six years).

**Figure 4:**
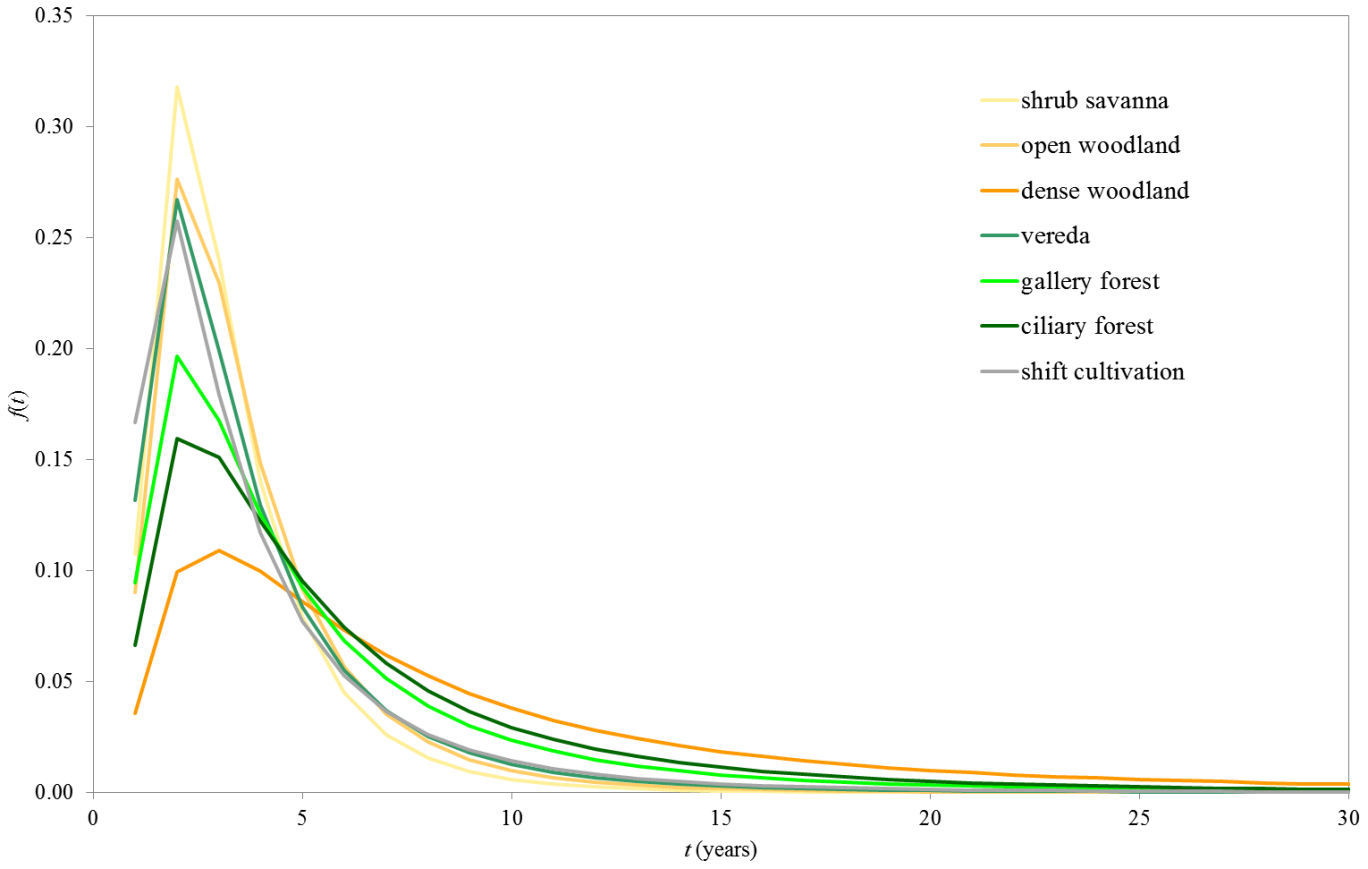
Fire interval distributions. Fire interval distributions (*f*) as a function of time since last fire (*t*) by land cover type for Jalapão State Park. Fire interval distribution is the probability that the time between consecutive fires is equal to *t*.

The mode corresponds to the peak in the fire intervals distributions, which occurs at year two, except for DW, which peaked at year three (Figure 4). The probability of having a two-year interval between consecutive fires was highest in SS (0.32), followed by OW (0.28), VR (0.27), SC (0.26), GF (0.20) and CF (0.16). For DW the probability of having a three-year interval between consecutive fires was 0.11 (lowest peak value). Fire interval distributions can be aggregated in three groups according to the shape of the curves: SS, OW, VR, and SC, with probability higher than 0.26 at age two, falling abruptly to low values at older ages (e.g. 0.08≤ *f*(5) ≤0.09 and 0.02≤ *f*(8) ≤0.03); GF and CF with probability of fire between 0.16 and 0.20 at age two, and a less sharp decline of the curve (e.g. 0.09≤ *f*(5) ≤0.10 and 0.04≤ *f*(8) ≤0.05); DW, with a smooth decline (*f*(3) = 0.11, peak value; *f*(5) = 0.09; and *f*(8) = 0.05).

**Figure 5:**
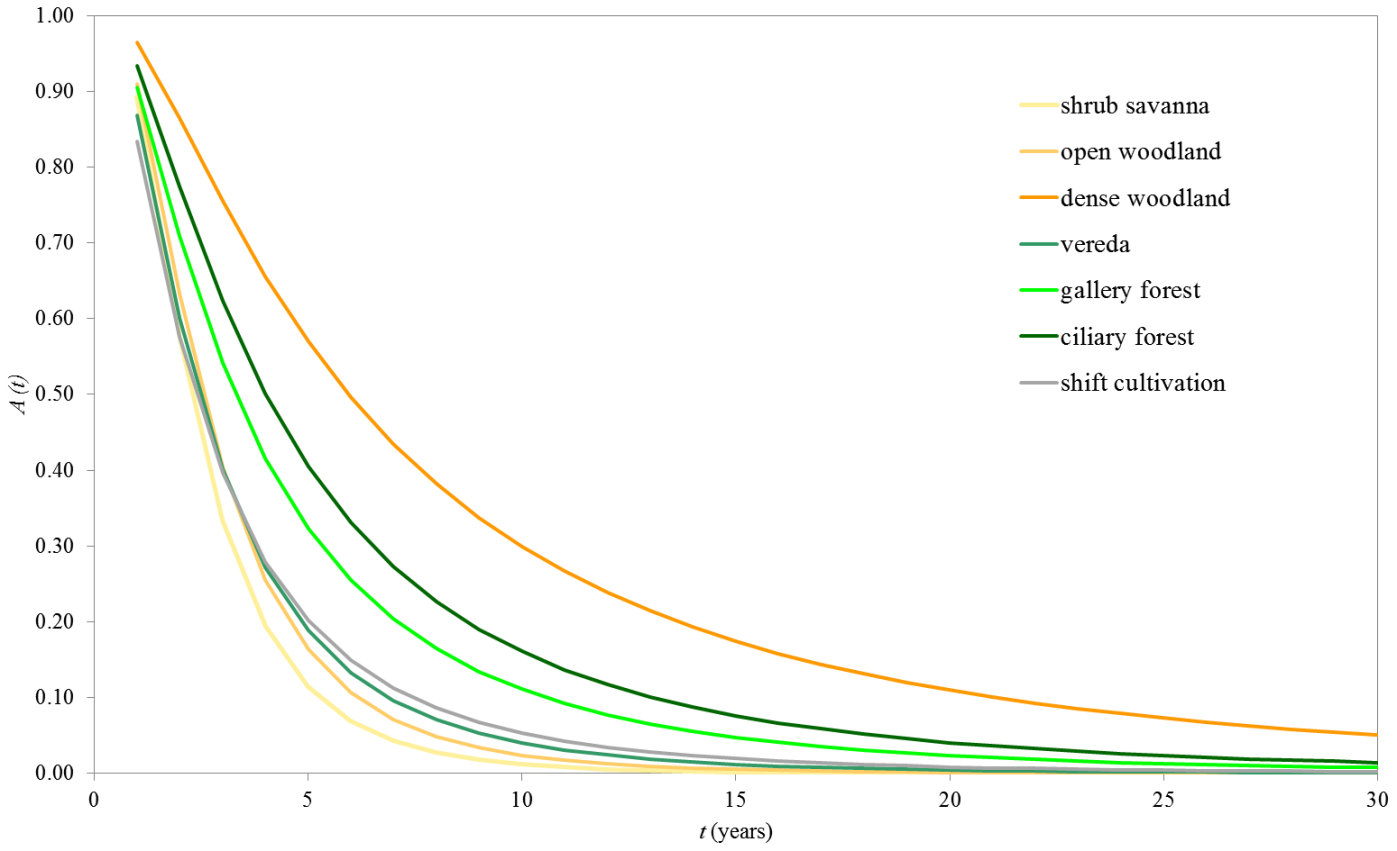
Survival distributions. Survival distributions (*A*) as a function of time since last fire (*t*) by land cover type for Jalapão State Park. The survival distribution is the probability of any given vegetation patch having survived fire for longer than *t*.

Survival distributions (Figure 5) have high values during the first year for all land cover types (*A*(1) ≥0.83), decreasing more or less abruptly, depending on land cover type: SS, OW, VR and SC, with curves falling abruptly (e.g. 0.11≤ *A*(5) ≤0.20 and 0.01≤ *A*(10) ≤0.05); GF and CF, with curves showing a less sharp decay (e.g. 0.32≤ *A*(5) ≤0.41 and 0.11≤ *A*(10) ≤0.16); DW, with the lowest decay rate of all curves (e.g. *A*(5) = 0.57 and *A*(10) = 0.30). The survival distributions indicate, for instance, that less than 10% of the landscape survives unburned up to year seven in SS, OW, VR and SC, up to year 10 in GF, up to year 13 in CF, and up to year 20 in DW.

**Figure 6:**
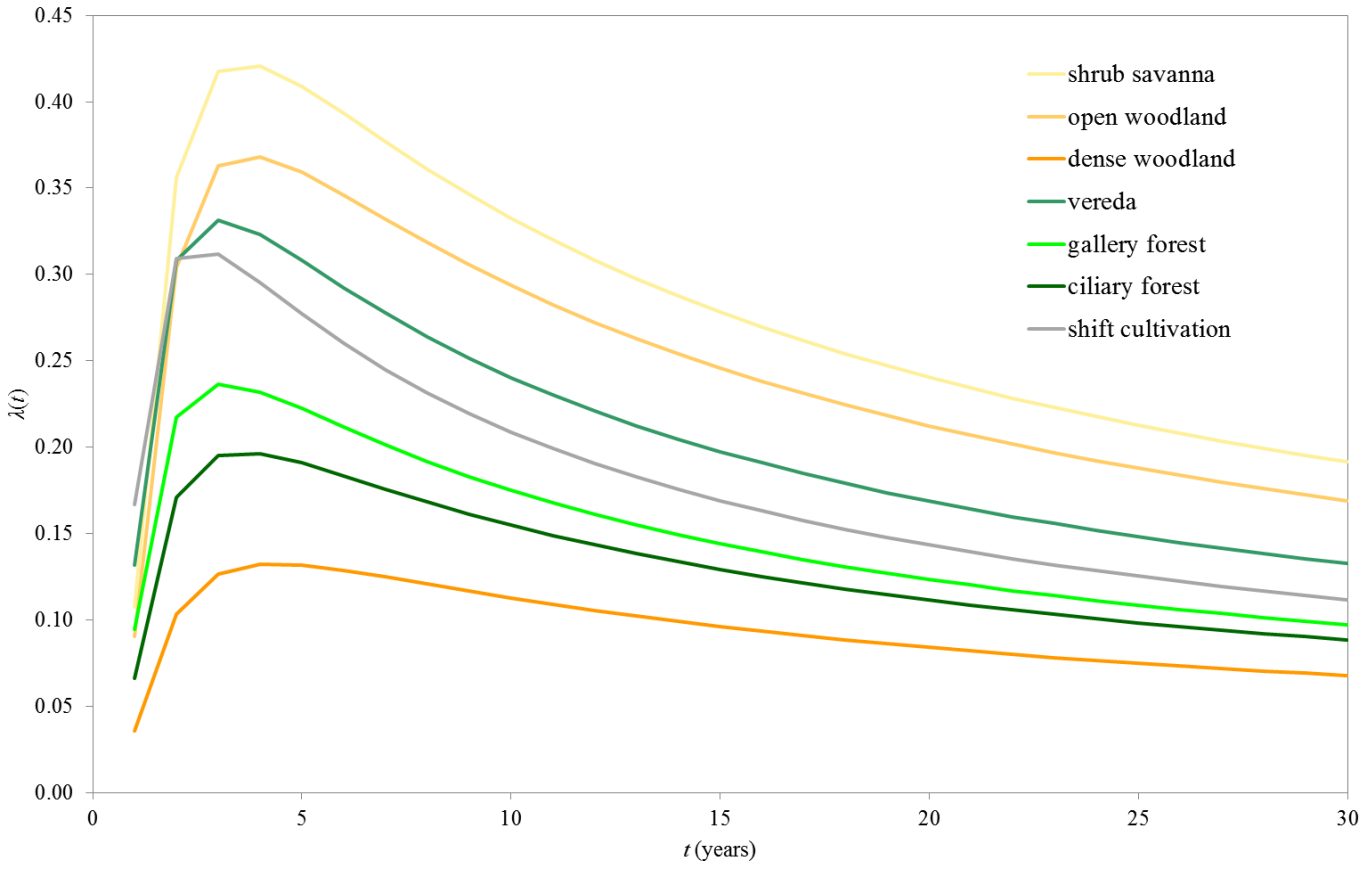
Hazard of burning distributions. Hazard of burning distributions (*λ*) as a function of time since last fire (*t*) by land cover type for Jalapão State Park. The hazard of burning distribution is a conditional probability of burning during time interval *t*, given survival up to that point.

Hazard of burning functions initially increase, reaching a peak at years three or four since the last fire (turning point), and then gradually decrease (Figure 6). The same three groups of land cover type can be identified, although there are differences in the turnings points within the same group: SS, OW, VR and SC with the highest probabilities of burning, given survival up to that point (hazard peak values of 0.42 and 0.37 in year four, and 0.33 and 0.31 in year three, respectively); GF and CF, with intermediate probabilities of burning (0.24 peaking at year three and 0.20 peaking at year four, respectively); DW, with the lowest probability of burning (hazard peak value of 0.13 at year four).

There was a negative relationship between hazard of burning and FRP. Sorting hazard of burning functions by ascending order we obtained, for every *t*, the following: *λ*_DW_(*t*)<*λ*_CF_(*t* )< *λ*_GF_(*t* )< *λ*_SC_(*t* )< *λ*_VR_(*t* )< *λ*_OW_(*t*)<*λ*_SS_(*t*) which is exactly the same order when sorting FRP values by descending order. Land cover types with the highest FRP are the ones with the lowest conditional probabilities of burning as a function of time since last fire.

## Discussion

### Burned area mapping and fire rotation period

The highest fire incidence in JSP was observed in SS and OW, physiognomies supporting large fuel loads forming a continuous fuel bed, where the grass and litter cure rapidly at the beginning of the dry season due to the low canopy cover. In these areas, cattle ranchers use fire every couple of years to stimulate grass regrowth for pastures. The lowest incidence of fire occurred in DW, where grassy fuels are highly discontinuous and fuel moisture tends to be higher, due to denser canopy cover. Anthropogenic fire is scarce in large areas of rocky substrate with rugged relief, which occur in 83% of DW in JSP. Intermediate values of fire incidence for VR and SC are likely due to burning practices to extract products directly from managed areas (use of fire to stimulate golden-grass flowering in VR, or to burn for clearing purposes in SC). Fire incidence was low in CF and GF because they are closed forest formations in humid habitats along rivers, and the fuel remains very moist throughout the year. In GF fire incidence was higher than CF because it is usually found in areas adjacent to VR, where the absence of fire breaks leads to fire spreading.

Fire return period of three years for JSP (APAB = 33%) is similar to the values observed in western Arnhem Land (WAL), a savanna region in northern Australia, where FRP varied between 2.7 and 2.8 years (APAB 36%–37%) [24], [36]. [60] found higher APAB in Litchfield National Park (56%) and Nitmiluk National Park (41%), in northern Australia. On the other hand, [61] found lower APAB in Kakadu National Park, an area adjacent to WAL, with values ranging from 21% to 27% in sandstone habitats. Both Litchfield National Park and Kakadu National Park are protected areas and tourist attractions, like JSP. Controlled burning is practiced at the national parks in the coolest months to prevent severe and extensive fires in the hottest months. Western Arnhem Land is under aboriginal tenure, and traditional owners have been using fire as a land management tool for thousands of years, for ‘cleaning up’ the country and facilitate walking, hunting, and to encourage growth of plant and animal foods. In general, there were also similarities between fire frequency in the different physiognomies of JSP and those from northern Australia, reported by [36]. In both studies the fire incidence was higher in woodland and herbaceous-shrubbery physiognomies (SS and OW for JSP and woodlands and open forests in WAL). SS and OW had lower APAB values (37% and 36%, respectively) compared to woodlands and open forests (45% and 42%, respectively), but GF and CF (23% and 22%, respectively) had very similar values to closed forest (20%), in Australia. This highlights the strong similarities between these two tropical savannas and justifies analysis by land cover type.

### Fire frequency analysis

Fire interval distributions reveal that a large fraction of the landscape burns every two to three years, and fire survival distributions reveal that the extent of vegetation surviving this period and remaining available for burning at a later age decreases abruptly. Hazard of burning functions reach a peak three to five years after a fire and then decrease, meaning that because a large fraction of the landscape burns two to three years after the previous fire, the extent of landscape surviving this period and remaining available for burning at an older age decreases sharply. Thus, the probability of fire occurring in an older fuel age class is lower than that of it occurring in a younger class, mainly because long unburnt patches are quite rare in these landscapes. Although the decrease in the hazard of burning function does not imply a reduction in fuel loading, there is evidence that fuel loads in tropical savannas attain maximum values three to six years after a fire and then decrease [62], [63]. Hazard of burning is also correlated with FRP since land cover types with the highest probabilities of burning are also the ones that burn more frequently, and need less time to burn an area equivalent to the study area.

Fire interval analysis indicated also clear patterns between physiognomies with low and high canopy cover, and riparian physiognomies. A short fire return interval (three years) was associated with physiognomies with lower density of trees (SS, OW and VR), where continuous fuels and geomorphologic features easily allow fire spread, and SC, where fire is used for crop residue burning and land clearing. Slightly longer fire return intervals (four or five years) were observed in riparian physiognomies (CF and GF) whereas longer fire return intervals were observed in DW, a high tree density physiognomy. These values also showed similarities with the median fire intervals estimated at WAL, Australia [36]; SS, OW, VR and SC had the same intervals as sandstone woodlands in WAL, and GF and CF approximately the same intervals as closed forests in WAL.

### Fire ecology and management

According to our results, fires with a median fire interval of three to six years have been occurring since 1997 at JSP, a protected area of Cerrado. Those fires require large efforts and resources to be controlled, and demonstrate the ineffectiveness and inadequacy of the total fire suppression policy imposed by the Brazilian government agencies. Although the Cerrado is a fire-prone ecosystem and plants and animals show adaptations to frequent fires [17], frequently burned Cerrado areas tend to become more open and grassy [2,2464,]. There is also strong evidence that fire-sensitive vegetation (e.g. *Blepharocalix salicifolius, Sclerolobium paniculatum*) are in decline under the present-day Cerrado fire regime [6], [17], [65], in agreement to what is happening in other tropical savanna regions [66]. One exception is the golden-grass, in VR. Based on empirical knowledge of indigenous people, [42], [67] verified that bi- or tri-annual fires promote the optimum population growth of golden-grass yet longer intervals do not diminish its population. With this in mind, [67], [68] suggested joining both traditional ecological knowledge and conservation-oriented management, to extend current fire intervals. A similar strategy is being implemented in Australian tropical savannas, where indigenous aboriginal knowledge is incorporated into environmentally sustainable fire management [66].

Future fire management scenarios for JSP, including those that assume the absence of resident populations, will be able to use results of this study as a baseline against which to identify the need for modification of current fire regimes, and assess the effectiveness of subsequent interventions. Since our analysis was broken down by major vegetation type, it can support the development of specific management strategies, geared towards enhancing the ecological values that justify the protected status of this area. For example, landscape formations with short fire return intervals, such as SS, OW, and VR (which occupy 83% of the JSP burnable area), more than a change in overall fire frequency, may benefit from a shift in the timing of burning towards earlier in the season, to reduce the severity of fire effects, including the amount of pyrogenic emissions of greenhouse gases [69]. On the other hand, landscapes with longer return intervals and vegetation that is more fire-sensitive, such as GF, CF and DW [68], probably ought to be the focus of efforts to contain escaped anthropogenic fires.

## Conclusions

Fire frequency in JSP is very high, but not uncommon for tropical savannas, with median fire return intervals of three to six years, depending on the vegetation type. This high fire incidence is a function of the markedly seasonal climate of the Cerrado, fuel biomass accumulation, and traditional use of fire to stimulate the growth of palatable grasses for cattle, or to stimulate golden-grass flowering, used in local handicrafts. The probability of burning as a function of time since last fire reaches an early peak between three to four years, and subsequently declines due to fuel dynamics, reflecting a strong fuel age-dependency only in young vegetation patches. Open savanna physiognomies, that occupy most of the area of JSP, and shifting cultivation areas display the shortest fire intervals (median value of three years), and dense woodland formations the longest (median value of six years). Riparian physiognomies have intermediate median fire intervals of four to five years. The discrete lognormal model is appropriate for fire frequency analysis in tropical savannas, because it fits well regimes with short fire intervals, producing essential data for fire management, biodiversity conservation and greenhouse gas emissions estimates.

Sustainable management in protected areas of Cerrado should be a continuous process, incorporating scientific knowledge and traditional ecological knowledge of indigenous people and local communities [17], [70]. Examples from countries such as Mali and Madagascar show that attempts to impose regulations and legal restrictions rarely contribute towards solving the savanna fire management problem. On the contrary, such rules often undermine traditional practices and weaken local land use management, including the capability for pro-actively managing a fire regime [71]. Ecologically sustainable management of fire regimes in Cerrado protected areas requires resources, time, planning capacity, and knowledge, and ought to be developed on a solid scientific foundation, and aim at enhancing the environmental values they are meant to protect.
